# Distance decay in delivery care utilisation associated with neonatal mortality. A case referent study in northern Vietnam

**DOI:** 10.1186/1471-2458-10-762

**Published:** 2010-12-13

**Authors:** Mats Målqvist, Nazmul Sohel, Tran T Do, Leif Eriksson, Lars-Åke Persson

**Affiliations:** 1 International Maternal and Child Health (IMCH), Department of Women's and Children's Health, Uppsala University, Uppsala, Sweden; 2 National Institute of Nutrition (NIN), Ministry of Health, Hanoi, Vietnam

## Abstract

**Background:**

Efforts to reduce neonatal mortality are essential if the Millennium Development Goal (MDG) 4 is to be met. The impact of spatial dimensions of neonatal survival has not been thoroughly investigated even though access to good quality delivery care is considered to be one of the main priorities when trying to reduce neonatal mortality. This study examined the association between distance from the mother's home to the closest health facility and neonatal mortality, and investigated the influence of distance on patterns of perinatal health care utilisation.

**Methods:**

A surveillance system of live births and neonatal deaths was set up in eight districts of Quang Ninh province, Vietnam, from July 2008 to December 2009. Case referent design including all neonatal deaths and randomly selected newborn referents from the same population. Interviews were performed with mothers of all subjects and GIS coordinates for mothers' homes and all health facilities in the study area were obtained. Straight-line distances were calculated using ArcGIS software.

**Results:**

A total of 197 neonatal deaths and 11 708 births were registered and 686 referents selected. Health care utilisation prior to and at delivery varied with distance to the health facility. Mothers living farthest away (4^th ^and 5^th ^quintile, ≥1257 meters) from a health facility had an increased risk of neonatal mortality (OR 1.96, 95% CI 1.40 - 2.75, adjusted for maternal age at delivery and marital status). When stratified for socio-economic factors there was an increased risk for neonatal mortality for mothers with low education and from poor households who lived farther away from a health facility. Mothers who delivered at home had more than twice as long to a health facility compared to mothers who delivered at a health care facility. There was no difference in age at death when comparing neonates born at home or health facility deliveries (p = 0.56).

**Conclusion:**

Distance to the closest health facility was negatively associated with neonatal mortality risk. Health care utilisation in the prenatal period could partly explain this risk elevation since there was a distance decay in health system usage prior to and at delivery. The geographical dimension must be taken into consideration when planning interventions for improved neonatal survival, especially when targeting socio-economically disadvantaged groups.

## Background

There has been an increasing awareness that the perinatal period is a neglected area in recent years, and interventions targeting mothers and newborns have been encouraged [1]. Nearly four million newborns die during the first four weeks of life every year [2,3] and the rate of neonatal mortality has remained basically unchanged in the past decades [4]. Some improvements can be seen, but still the pace is slow, especially in the early neonatal period [5]. Most of these neonatal deaths occur during the first day of life and complications related to delivery care make up a large proportion of the overall neonatal mortality [3,6]. Skilled assistance at delivery and access to emergency obstetric care are the most effective interventions to prevent these early and intra-partum related deaths [7]. This requires both the availability of such services as well as the will and possibility for pregnant women to seek this care at delivery [8].

Thaddeus and Maine's conceptual framework of the three delays in care seeking has been widely used when investigating health care utilisation [9]. They developed their thoughts around obstetric emergencies, but the framework is valid for any kind of care seeking behaviour, and Gabrysch and Campbell adapted it to also cover use of preventive services around delivery [8]. Geographical factors such as distance between home and health institutions are part of the first and the second delay and suggested an influence on the choice of delivery place [8] as well as being related to neonatal mortality risks [10]. It has also been demonstrated that the usage of health services decreases with increasing distance between health facilities and families' homes. This phenomena, often labelled by the geographical term distance decay [11], has been used to describe various situations and patient groups [12-14]. It has also been shown for the utilisation of maternal health care and delivery services [15-17]. Geographical Information Systems (GIS) can be used to investigate spatial dimensions of different health outcomes. This has been applied to study infectious diseases such as malaria and acute respiratory infections [18,19], as well as to study geographical variation in child mortality [20]. So far little has been explicitly done in the field of peri- and neonatal health.

We have initiated a cluster-randomised trial for improved neonatal health and survival in the Quang Ninh province in northern Vietnam. The trial that has been given the acronym NeoKIP (Neonatal Health - Knowledge Into Practice, ISRCTN 44599712), is a collaboration between the Ministry of Health, Uong Bi General Hospital and Hanoi School of Public Health in Vietnam and Uppsala University in Sweden. Preliminary results from the initial part of the trial indicate that ethnic group and health system utilisation before and at delivery were major determinants of neonatal survival [21]. We have also shown a strong negative association at baseline between home delivery rate and the chances of neonatal survival [22] and that a quarter of the mothers losing their baby in the neonatal period did not have any contact with the health system prior to death [23]. In this study we further investigate the determinants of neonatal mortality, including the care seeking behaviour at delivery by the addition of GIS data and analysis techniques. Specifically, we aim to examine the association between distance from the mother's home to the closest health facility and neonatal mortality. In this analysis a special emphasis will be put on different patterns of perinatal health care utilisation.

## Method

### Setting

**Figure 1:**
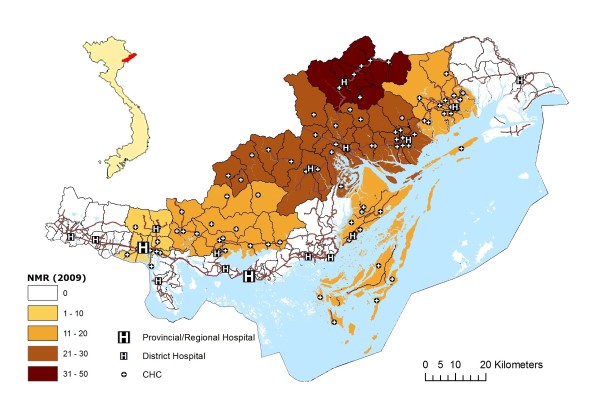
**NeoKIP study area in Quang Ninh province in northern Vietnam**.

Quang Ninh province is located in the north-east corner of Vietnam, right on the border to China (Figure 1). Demographically there are approximately one million inhabitants divided into ten different ethnic groups, with the hegemonic group of Kinh being the largest. The terrain is heterogeneous with mountainous areas in the inlands and in the north and flatlands in the south and along the coastline. The coastline is extensive and the archipelago outside Ha Long City is a place both for tourism and a flourishing marine industry. Quang Ninh province also harbours a large mining industry and is a major exporter of coal. Like the rest of Vietnam the area is going through an economic transition with an annual growth rate of about 8% [24], potentially leading to increasing inequities in society since it will take time until the most remote areas are modernised.

Eight districts in the province with the highest neonatal mortality rate (>15/1000 in 2005) were chosen as the NeoKIP study area [25]. These districts are divided into ninety (90) communities, each with a health centre (CHC) and in each district there is a district hospital. Each CHC has a number of village health workers (VHW) employed to provide preventive care at village level, and in general the coverage of health workers is good [22]. It is also assigned to the VHWs to keep records of and report reproductive health events from their area of responsibility once a month to the midwife at the CHC. There are two tertiary hospitals in the province, one in Ha Long City, which is outside the study area, and one in Uong Bi district (Figure 1). The latter also functions as a regional hospital, serving neighbouring provinces. Mothers are free to seek antenatal care and delivery services at all levels of the health system and are not bound to their community of origin. In addition to the governmental health facilities there are hospitals run by the coal mining industry as well as a plenitude of private health care providers providing antenatal care. There are however no private providers of delivery care. A health insurance system covers maternal and child health services. Around half of the population is however not included in the health insurance system, but the government covers health care costs for children <6 years and for others who are classified as poor. The cost of delivery varies considerably between different service facilities [22]. The coverage of ambulance services is concentrated in the major hospitals and transportation for referrals is therefore mainly for the families themselves to arrange, even in emergencies.

### Study design and data collection

In order to identify subjects for the study, a team of trained data collectors recorded all births within the study area from July 2008 until December 2009. Data collection was performed by these data collectors through monthly visits to all CHCs where data were excerpted from records and staff and village health workers were interviewed for any missing information on births and deaths in the communities. All district level hospitals in the province, the provincial hospital in Ha Long and the regional hospital in Uong Bi were also visited monthly and information was excerpted from the records. Names and addresses of all mothers of newborns were recorded and entered into the study data base. The health status of all newborns was ascertained at one month after delivery and neonatal mortality cases were recorded. To avoid duplication of data, cross-checking between different data sources was performed and supervised by an assigned supervisor. All incident neonatal deaths were noted as cases. All live births were entered into a data base and once a month all new entries were grouped and assigned a number. By the use of a random number generator 6% from each monthly batch were selected as referents to ensure at least a 2:1 ratio between referents and cases.

Mothers of all cases and referents were interviewed at home eight to ten weeks after delivery, using a semi-structured interview form. Informed consent was obtained verbally by the interviewer.

### Geographic information

Information about geographical features (roads, hydrography, elevation and administrative boundaries) was accessed from the VidaGIS data base http://www.vidagis.com. VidaGIS provides data in the Universal Transverse Mercator (UTM) projection system. Global positioning system (GPS) was used to identify all health facilities and homes of cases and referents. Locations obtained using a GPS device (Garmin GPS 60 or Garmin GPS 60Cx) were transformed into the UTM projection system to calculate distance between homes and health facilities and entered into the study data base. Linear distances between homes and the closest health facilities at each level of the health care system were calculated using the "proximity" function in ArcMap 9.3. For mothers who delivered at a health facility total linear distance travelled from home to final place of delivery was calculated. Information about the place of first contact with the health system at time of delivery was used to calculate this distance using the "near" function in ArcMap 9.3. In case of intrauterine transfer within the health system linear distances were calculated between health facilities and added to the initial distance in order to get total distance travelled before delivery. All distances mentioned are one-way distances.

### Data analysis

The total number of births and deaths within the study period were used to calculate the neonatal mortality rate and proportion of home deliveries for the study area. Births and deaths were then sorted by district in the study area and neonatal mortality rates for each district were calculated. All further analyses were based on case-referent data. Distances from home to the closest health facility were divided into quintiles. The first and second quintile were grouped and labelled "close" and the third to fifth quintile were grouped and labelled "remote". The percentage of facility use at delivery in each quintile group was displayed to demonstrate distance decay. Crude odds ratios (COR) of neonatal mortality were calculated for distance quintiles and background variables. Ethnic group was further classified as either being from the majority group of Kinh or from any of the minority groupings in the study area. Household economic status was calculated using an asset index, as described elsewhere [21]. Variables associated with exposure and outcome at a significance level of p < 0.10 were considered as potential confounders and included in a multivariate model.

Previously we have demonstrated that the ethnic group of the mother and to some extent also maternal educational level are determinants of neonatal death, while economic status was shown not to influence the risk of neonatal death [21]. It is well known that ethnic minorities in Vietnam live in the more remote and isolated areas [26,27] and that there are larger concentrations of poverty in rural areas [28]. Therefore, to further come to terms with possible confounding stratification according to socioeconomic factors was made.

Pearson's chi-square test and Mann-Whitney U test was used for group comparison. Statistical analyses and data handling were performed in Intercooled Stata 9 and SPSS 17.0. A p-value < 0.05 was considered significant.

### Ethical approval

Ethical approval for this study was obtained from the Ministry of Health in Vietnam and the Research Ethics Committee at Uppsala University, Sweden. The NeoKIP project has been approved and supported by the Provincial Health Bureau in Quang Ninh.

## Results

During the study period from July 2008 to December 2009 there were 11 708 live births and 197 neonatal deaths registered (NMR 17/1000). There was a marked geographical variation in neonatal mortality rate when calculated on district level, with higher rates in the remote and mountainous districts of the province (Figure 1). Home delivery rate for the whole study area was 9.9% (1155/11708). Most deliveries took place at a hospital (8691/11708), with the Vietnam-Sweden General Hospital in Uong Bi being the major delivery service provider with 3340 deliveries from the study area during the time period.

### Cases and referents

The 197 incident neonatal deaths were registered as cases, and 686 live births were randomly selected as referents from the total population of live births. Mothers of 183/197 (93%) cases and 599/686 (87%) referents were available and interviewed. Mean age for case mothers was 24.1 years as compared to 25.4 years for referent mothers (p < 0.001). There was no difference in parity between mothers of cases and referents (Mann Whitney, p = 0.39). Fifty six percent (102/183) of cases and fifty four percent (322/599) of referents were boys. GIS coordinates for respondents' homes were obtained for 180 cases and 597 referents.

### Distance to health facilities

Straight-line (Euclidian) distances between respondents' homes and the closest community health centre, district hospital and tertiary hospital were calculated using the GIS coordinates. Distances were not normally distributed. Most mothers lived closer to a CHC than to a hospital. The median distance between home and the closest health facility was 927 meters for referents and 1437 meters for cases (p < 0.001) (Table 1), with a range of 13 - 10 418 meters.

**Table 1:** Median straight-line distance (meters) from respondent's home to health facilities, overall and in groups based on delivery place and outcome in Quang Ninh province, Vietnam.

		Cases	Referents	p-value*
**Distance to closest health facility**	Health facility delivery	1 108	881	0.06
	Home delivery	2 574	1 819	0.13
				
	All	1 437	997	<0.001
				

**Distance to Community Health Centre**	Health facility delivery	1 143	972	0.07
	Home delivery	2 594	1 819	0.12
				
	All	1 440	1060	<0.001
				

**Distance to District Hospital**	Health facility delivery	5 543	5 634	0.23
	Home delivery	10 262	9 692	0.87
				
	All	7 242	6 386	<0.01
				

**Distance to Tertiary Hospital**	Health facility delivery	52 209	37 773	<0.01
	Home delivery	73 415	70 890	0.76
				
	All	63 419	41 635	<0.001

*Mann-Whitney U for comparison of groups (180 cases and 597 referents).

The place where mothers first sought care for delivery was noted for all respondents. Among mothers who chose a facility delivery there was no difference between cases and referents whether they had gone to the closest health facility or chose to go to a more distant health facility for delivery (p = 0.58). Cases and referents who delivered at a health facility travelled the same distance to get there (p = 0.862), with a median of 2204 meters for cases and 1927 meters for referents. There was also no difference in perceived travel time from home to health facility between cases and referents, with a median 10 minutes for cases and 15 minutes for referents (p = 0.63). Mothers who chose to deliver at home had a longer distance to all levels of the health system (Table 1). In the referent group, the median distance from home to the closest health facility was twice as long for mothers who delivered at home compared to mothers who delivered within the health care system, 1819 m vs 881 m (p < 0.001) (Table 1).

### Distance decay

**Figure 2:**
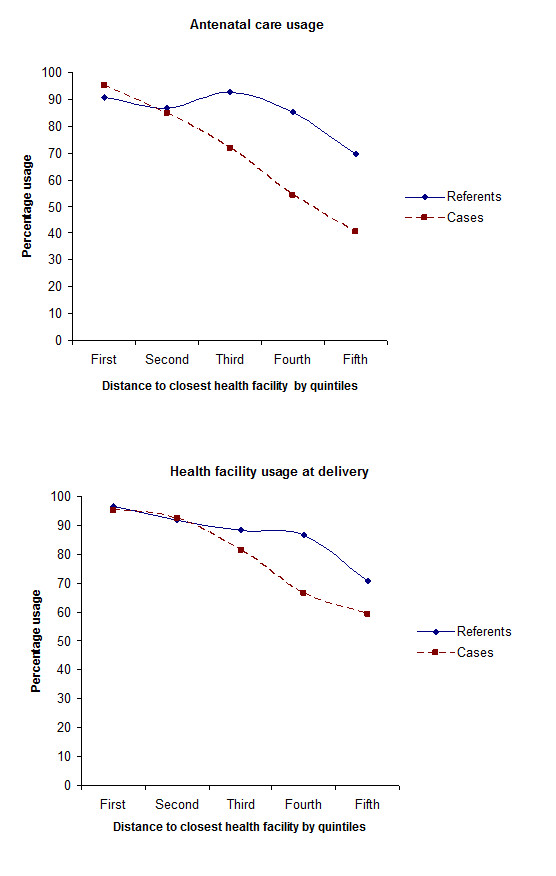
**Health facility usage during pregnancy and at delivery based on the distance from mother's residence to the closest health facility in Quang Ninh province, Vietnam**. (1^st ^quintile < 401 meters, 5^th ^quintile > 2233 meters).

Mothers of cases were less likely to deliver at a health facility (66.4%) compared to referent mothers (85.6%) (p < 0.001). Figure 2 demonstrates the relation between health facility utilisation during pregnancy and at delivery based on distance to the closest health facility, indicating a difference in distance-decay in perinatal care utilisation between cases and referents. Mothers of newborns who died in the neonatal period had a sharper decay in both attendance to antenatal care as well as in delivery at health facilities than referents.

### Neonatal mortality

**Figure 3:**
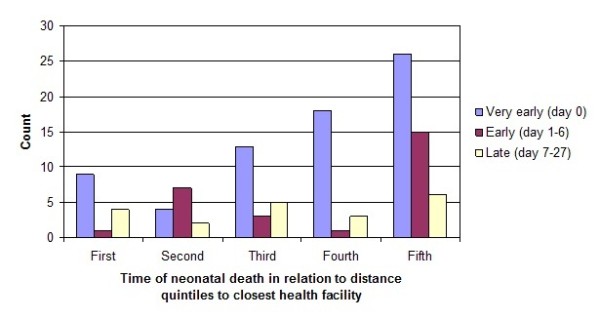
**Time of neonatal death divided by distance quintiles to closest health facility in Quang Ninh province, Vietnam, July 2008- December 2009 (χ^2 ^18.6, p = 0.017)**. 1^st ^quintile < 401 meters, 5^th ^quintile > 2233 meters.

Sixty percent (95/183) of the neonatal death cases died within the first 24 hours after delivery. There was an increasing proportion of very early neonatal deaths the farther away the mother lived from a health facility (Figure 3). There was however no difference in time of death when comparing home deliveries to health facility deliveries (p = 0.21). Neither did the place where mothers primarily sought care for delivery influence the time of death (p = 0.42) nor did the total distance from home to the final place of delivery (2007 meters for deaths on day 0 and 1879 meters for newborns dying during day 1-27, p = 0.75).

Crude odds ratios (COR) for factors associated with neonatal mortality are presented in Table 2. Sex of the newborn, mode of delivery, family structure and parity did not show any association with neonatal mortality (data not shown) and were not considered in a multivariate analysis (p > 0.10). Mother's marital status and age at delivery were associated on a significance level of 10%, and were included in further analysis (p < 0.10). Considering distance to the closest health facility to be in the casual pathway of the health care utilisation variables of antenatal care attendance and place of delivery, as expressed by the exhibited distance decay, these variables were also excluded from the multivariate analysis. After relevant adjustments in a multivariate logistic regression model there was an almost double risk of neonatal mortality for families living farthest away from a health facility (4^th ^and 5^th ^quintile, ≥ 1257 meters) compared to the group who resided closest to a health facility (1^st ^to 3^rd ^quintile < 1257 meters) (OR 1.96, 95% CI 1.40 - 2.75, adjusted for maternal age at delivery and marital status).

**Table 2:** Association between factors of the mother, the household and the health system and neonatal mortality (bivariate analysis) in Quang Ninh province, Vietnam.

	Cases (n)	Referents (n)	Crude odds ratio	Confidence interval (95%)
***Socioeconomic determinants***				
**Maternal ethnicity**				
Kinh	371	68	Ref	
Minority	228	115	2.75	1.94 - 3.91
				
**Maternal education**				
Tertiary school or higher	40	207	Ref	
Secondary school	39	151	1.33	0.82 - 2.18
Primary school	37	134	1.43	0.87 - 2.35
No schooling	65	106	3.17	1.97 - 5.09
				
**Household economy**				
5th quintile (rich)	12	120	Ref	
4^th ^quintile	22	120	1.83	0.86 - 3.89
3^rd ^quintile	23	113	2.04	0.96 - 4.31
2^nd ^quintile	60	124	4.84	2.41 - 9.71
1^st ^quintile (poor)	66	122	5.41	2.69 - 10.9
				
***Proximate determinants***				
**Distance to closest health facility**				
1^st ^quintile (<401 m)	30	119	Ref	
2^nd ^quintile (402 - 740 m)	26	120	1.28	0.68 - 2.44
3^rd ^quintile (741 - 1256 m)	32	119	1.60	0.86 - 2.97
4^th ^quintile (1257 - 2232 m)	48	120	2.38	1.32 - 4.29
5^th ^quintile (>2233 m)	54	119	2.70	1.50 - 4.85
				
Close (1^st ^to 3^rd ^quintile)	78	358	Ref	
Remote (4^th ^to 5^th ^quintile)	102	239	1.96	1.39 - 2.75
				
**Antenatal care visits**				
Yes	113	507	Ref	
No	70	91	3.45	2.35 - 5.07
				
**Place of delivery**				
Regional hospital	37	152	Ref	
District hospital	75	277	1.11	0.72 - 1.73
CHC	13	90	0.59	0.29 - 1.18
Home	58	80	2.98	1.79 - 4.96
				
Facility delivery	80	519	Ref	
Home delivery	58	125	3.01	2.02 - 4.49

When stratifying by socioeconomic factors no association between distance to the closest health facility and neonatal mortality could be found for Kinh mothers, mothers with higher education and mothers who were better off economically (Table 3). There was however a risk elevation for mothers with low education (OR 2.59, 95% CI 1.16 - 5.82, adjusted for maternal age at delivery and marital status) and for mothers from poor households (2.84, 95% CI 1.30 - 6.21, adjusted for maternal age at delivery and marital status). Ethnic minority mothers displayed a near significant risk (Table 3).

**Table 3:** Risk of neonatal mortality related to distance from home to closest health facility, stratified by socio-economic variables, in Quang Ninh Province, Vietnam

		Cases (n)	Ref (n)	Odds ratio*	Confidence interval (95%)
**Household economy**					
**Non-poor**	Close	68	317	Ref	
	Remote	48	159	1.40	0.92 - 2.13
**Poor**	Close	10	41	Ref	
	Remote	54	80	2.84	1.30 - 6.21
					
**Mother's ethnicity**					
**Kinh**	Close	45	226	Ref	
	Remote	23	104	1.30	0.75 - 2.28
**Minority**	Close	33	92	Ref	
	Remote	79	135	1.61	0.99 - 2.62
					
**Maternal education**					
**Completed** **1° school (≥5 y)**	Close	68	325	Ref	
	Remote	46	165	0.84	0.86 - 2.01
**Not completed** **1° school (<5 y)**	Close	10	33	Ref	
	Remote	54	73	2.59	1.16 - 5.82

"Close" representing 1^st ^to 3^rd ^distance quintiles (<1257 m), "Remote" representing 4^th ^and 5^th ^quintiles.

* Odds ratios adjusted for maternal age and marital status at delivery.

## Discussion

In this study we have examined the association between distance to the nearest health facility and neonatal mortality and found an increased risk of neonatal death for mothers who live farthest away from health facilities. We have also shown distance decay in antenatal care attendance and facility usage at delivery. Mothers who delivered at home live farther away from a health facility than mothers who delivered within the health system and our results demonstrate how the rate of facility use at delivery decreases as the distance increases. This indicates that distance is a factor for the choice or necessity of home delivery and is in concord with previous research showing that an increasing distance from residence to the closest health facility decreases the delivery care utilisation rate [15,29,30]. Neonatal mortality was also associated with antenatal care use and the place of delivery. The increased risk for mothers living farthest away persisted when adjusting for place of delivery and antenatal care attendance. Health care utilisation patterns can thus not fully explain the association between distance and neonatal mortality. We also found that there was an earlier distance-decay effect among mothers who suffered a neonatal death than among referents (Figure 2) showing that there are other factors than distance affecting the choice of delivery place. One factor for the choice of delivery place not investigated here is the perceptions of the quality of health facility care. Earlier research has described a lower level of knowledge about perinatal health issues among health staff in the more remote and mountainous areas where distances in general are longer [31]. This could contribute to the demonstrated association between distance to the closest health facility and neonatal mortality.

When stratifying by the socioeconomic determinants the association between neonatal mortality and distance disappeared in the Kinh group and in the groups with higher education and better household economic status. It is well known that the people living in remote areas in Vietnam in general are more disadvantaged when it comes to economy and education [32,33]. There is also a higher density of ethnic minorities in the remote areas [26,28]. This association between socio-economic factors and distance has been taken as an argument many times for the disadvantaged position of these vulnerable groups [33]. Our results indicate that distance is also an important factor for neonatal survival among the least privileged, and may be an important piece in explaining the previously described increased mortality risk for newborns of mothers with low education and ethnic minority background [21,34]. Lack of resources for transportation and costs associated with facility delivery, poor understanding of the dangers of delivery and the importance of preventive delivery services might be factors hindering mothers in overcoming the obstacle of distance to health facilities.

This study is population based, including all neonatal deaths that occurred in the study area during 18 months. By applying a case-referent design we get a good sample to study determinants of neonatal mortality even if not all neonatal mortality cases were identified, since the referents represent the study population. However, we still believe that we have managed to capture and identify most neonatal deaths through the data collection system set up by the research group, not relying primary on faulty health records but on first hand information from local care givers. The primary limitation of this study can be found in the geographical measurement. As discussed above, distance is a difficult entity to pinpoint and it is up for discussion whether straight-line distances are really a trustworthy proxy for the true distance. Straight-line distance has however been frequently used in previous studies. Another limitation in relation to measuring distances is the diversity of geographical traits of the study area, which consists of both mountainous areas as well as flatlands and coastal areas. It can be argued that a straight-line distance in a mountainous area is not equal to the same distance in the flatlands. However, even this would rather strengthen the association between distance and neonatal mortality, since most neonatal deaths were found in the mountainous area, as depicted in figure 1.

We have used linear distances for our analyses. Two-dimensional road distances were manually measured using the ArcGIS software as well. These distances were proportional to straight-line distances with a factor of 1.4. Considering the imperfection of the concept of road distance we chose not to use them in the analysis. In real life there are a lot of components to consider when calculating the true distance, like slope, road quality and temporary obstructions along the road. There is also a tendency in any GIS program to underestimate the true length of a geographic line due to short-cutting and generalizations [35]. Straight-line distances are on the other hand well defined and calculated consequently and may act as a proxy for the true distance. Even if the true distance between home of the respondent and the closest health facility or the facility where delivery care was first sought would be refined by the use of more detailed geographical information, there would still be a question about transportation and other constraints on care seeking. The perception of physical accessibility, of which the true distance is one component only, plays an important part for the first delay in care seeking and the ability to pay and lack of transportation may for example play a crucial part in the second delay [8]. Shannon *et al *argue that what actually counts is the total effort made to reach a health facility and suggest that perceived travel time could be a good approximation of this effort [11]. In our data there is neither a difference in straight-line distance travelled nor in perceived travel time between cases and referents, further strengthening the use of straight-line distance as an approximation for geographical constraints on delivery care seeking.

The Vietnamese government has for many years prioritized primary health care and managed to achieve a good coverage of health facilities. This is reflected in our results by the relative short distances to a health facility for most of the mothers. Despite this, we can show that there is an association between the distance to the closest health facility and neonatal mortality. To further expand the numbers of CHCs, however, does not seem like a viable option considering the already short distances in this setting. The quality of roads might be an area where improvements may affect facility delivery rates. However this is usually beyond the scope of the health care system. In other settings, where travel time to the health system is long, trials with maternity waiting homes (MWHs) have been made. A Cochrane review has however concluded that there is not enough evidence to support the effectiveness of such facilities [36].

## Conclusion

Distance from home to the closest health facility was associated with neonatal mortality risk. Delivery care utilisation could partly explain this risk elevation since distance was an important factor for the decision to deliver at a health facility. To mothers who were poor, had low education and belonged to an ethnic minority distance to the closest health facility was an important determinant for neonatal survival. Mothers, especially from socioeconomically disadvantaged groups, must therefore be facilitated to overcome the constraints of distance and encouraged to deliver at a health facility.

## Competing interests

The authors declare that they have no competing interests.

## Authors' contributions

MM had the primary responsibility in all steps of the study and supervised field work together with TTD and LE. MM, LE and LÅP developed the study design and analyzed data together with NS. All authors were involved the writing of the manuscript and have approved the final version for publication.

## Pre-publication history

The pre-publication history for this paper can be accessed here:

http://www.biomedcentral.com/1471-2458/10/762/prepub
